# A comparative analysis of primary school meal nutrition across the low- and high-poverty boroughs of Inner London

**DOI:** 10.1186/s40795-026-01280-w

**Published:** 2026-03-18

**Authors:** Sophie Ognjenovic, Honglin Dong

**Affiliations:** 1 School of Health and Medical Sciences, City St George’s, University of London, London, UK; 2 Department of Nutritional Sciences, King’s College London, London, UK

**Keywords:** School meals, Nutrition, Childhood obesity, Socioeconomic status, London

## Abstract

**Background:**

Socio-economic status is a known predictor of childhood obesity. Improvements to school meals have been promoted as a method of combating rising childhood obesity rates, especially in low-income populations. However, little is known about how school food offerings differ across areas of low and high socio-economic status. This study aims to examine differences in school lunch nutrition across socioeconomic strata and compare these differences to small-scale regional childhood obesity prevalence.

**Methods:**

This was a cross-sectional study of electronically published school lunch menus and a longitudinal analysis of UK National Child Measurement Programme data. Participants were a randomly selected sample of free, state-funded primary schools with 200–399 pupils within the two highest and two lowest child-poverty boroughs of Inner London, UK (*n* = 20), along with borough-level data on child BMI in reception and year 6 (*n* = 4). School meals were evaluated for nutrient content using Nutritics and for objective healthiness using the Nutrient Profiling Model.

**Results:**

Lunches in high-poverty boroughs were significantly lower in total energy, carbohydrates, fat, and sugars (*p* < 0.001), but lower in iron, zinc, and Vitamin A (*p* < 0.01) compared with the most affluent areas. Using the nutrient profiling model, meals in high-poverty boroughs scored significantly better in both main courses (mean difference = -0.53, *p* = 0.016) and desserts (mean difference = 5.50, *p* < 0.001).

**Conclusions:**

Overall, meals in high-poverty boroughs were more nutritious than those in the most affluent areas, though they were lower in some key micronutrients. Despite this, rates of overweight, obesity, and severe obesity are higher in these boroughs, indicating that factors other than school food nutrition may play more crucial roles in the relationship between socio-economic status and childhood obesity.

**Supplementary Information:**

The online version contains supplementary material available at 10.1186/s40795-026-01280-w.

## Background

The rising prevalence of childhood obesity in recent years has become a public health concern worldwide. In England, nearly one in four children entering year 6 in 2023 were obese or severely obese [1]. Combined with overweight, children with higher than optimal BMI make up 35.9% of the total population between the ages of 5 and 11 years [2]. This high prevalence poses significant implications for the well-being of future generations, given the numerous health concerns associated with childhood adiposity [3].

Socioeconomic status (SES) is a known predictor of childhood obesity and diet quality. Lower SES is linked to reduced fruit and vegetable intake, and increased consumption of fat, refined sugar, and overall malnutrition [4]. As a result, low-income children are more likely to be obese than those in high-income families [5]. These effects are also evident at the regional level: areas with lower SES show higher childhood obesity rates in England [6]. According to the 2023 National Child Measurement Programme (NCMP), children in the most deprived regions were over twice as likely to be obese upon entering year 6 compared to those in the least deprived areas [7]. At the local authority level, regions with the highest child poverty had 6.9% more overweight and obese children than those with the lowest poverty rates [8].

Schools play a key role in shaping children’s diets [9], and UK policies have promoted school meal uptake over packed lunches, citing a general lack of nutritional adequacy in packed lunches as motivation to consume school food [10]. In 2023, the Mayor of London passed legislation providing universal free school meals at all state-funded Greater London primary schools [11], thus further encouraging school meal take-up. As of 2024, 59% of all English children and 73% of children eligible for free meals consume school food more than 4 times per week [12]. Improvements to school lunch nutrition standards and expansion of free school meal eligibility have the potential to positively impact childhood adiposity rates. In the United States, the 2010 Healthy, Hunger-Free Kids Act (HHFKA) which expanded free school lunch eligibility and improved nutritional standards for food served in schools was shown to reduce BMI z-scores and improve healthy-weight prevalence, particularly among low-income children [13]. Some UK studies show reductions in BMI after policy changes [14], others report no improvements in diet quality [15].

Effective school meal interventions for childhood obesity require nutritionally adequate food offerings. While school meals have been seen to be healthier than packed lunches [16], it has also been found that over half of the calories in English primary school lunches come from ultra-processed foods [17]. UK school meals have also been found to be above the recommended values for sugar and salt [18] and below the recommendations for iron and calcium [16].

Food served in English state-funded schools must meet the School Food Standards set by the Department for Education [19]. These guidelines detail mandatory meal components such as daily fruit and vegetables, starchy foods, dairy, meat (at least three times a week), and oily fish (once every three weeks), alongside limits on high-fat, high-sugar, and high-salt items [20]. However, adherence to the School Food Standards remains a persistent issue. One study found that English secondary schools met only 64% of these standards on average, with the lowest compliance being in energy-dense, high-fat, and high-sugar foods [21]. Another study estimated that just 25% of schools fully comply with the School Food Standards Practical Guide [22]. Although calcium, iron, and zinc are listed as nutrients of concern [20], school meals often fail to meet recommended levels [16]. Currently, there is no national system for monitoring school meal quality in England [22]. Without proper monitoring and enforcement of school food standards, the expansion of free school meal eligibility and the promotion of school meal uptake have the potential to do more harm than good on the childhood obesity front.

Limited information is available on how school food offerings differ across socioeconomic strata. In London, decisions on what food is served in schools are left to headteachers or, in some cases, the local authority councils [10]. These officials may then choose to have catering in-house or outsource to a contract caterer depending on factors such as cost, staffing, and training investment [10, 23]. In-house caterers are generally more cost-effective; however, this comes with challenges such as a greater overhead for the wider school team, a need for investing in external training for chefs, and a potential lack of innovation or nutritional expertise [23]. This decentralised approach to meal provision gives way to the potential for variations in meal nutritional quality depending on a school’s location or postcode due to budget, available talent, or a school’s staff availability. At present, there are no studies examining regional differences in school meal menus across London or any other major city.

The primary aim of the study is to determine whether there are significant differences in the nutritional composition of school meals between the low-poverty and high-poverty boroughs of Inner London. The secondary aim is to analyse the prevalence of childhood overweight and obesity in these boroughs and explore the relationship between the nutritional quality of school meals and the rates of obesity to assess whether disparities in school food provision may be contributing to health inequalities among children in Inner London.

## Methods

This is a secondary data analysis consists of two components: nutritional analysis of school lunch menus (without recipes) provided online in the most and least deprived boroughs within the 12 “inner borough of London, UK [24] and an analysis of childhood obesity prevalence in these boroughs from reception to year 6 using BMI data from the National Child Measurement Programme (NCMP) [2]. Boroughs were selected using Trust for London’s 2023 Poverty Profile [25]; the two boroughs with the highest child poverty rates and the two boroughs with the lowest child poverty rates were selected for inclusion in the study. Demographic, economic, and household data for the four boroughs were sourced from Trust for London’s borough profiles [26] and the Office for National Statistics’ local indicators, based on the 2021 Census [27].

### School selection

**Fig. 1:**
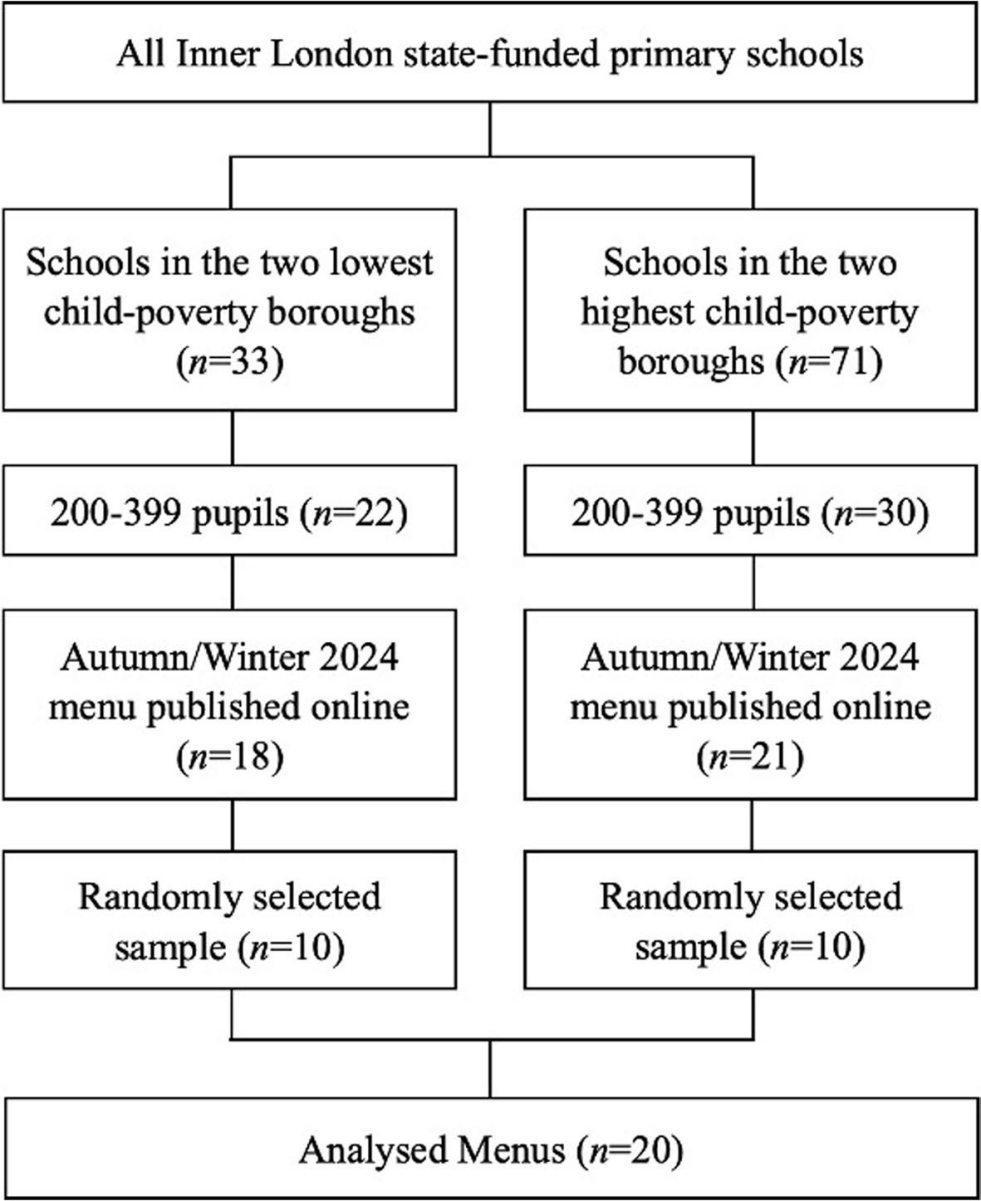
School meal menu selection process

Twenty state-funded primary schools, five per borough, were randomly selected from the UK government’s database of 2024 open state-funded schools [28]. Pupil counts for the 2024–25 school year were individually identified by searching the UK government register of schools in England [29]. All state-funded primary schools in the four examined boroughs with 200–399 pupils (*n* = 52) were screened for eligibility by searching their public websites for Autumn/Winter 2024 lunch menus. From the 39 schools within the target population that had menus published online, five were randomly selected within each of the four boroughs (*n* = 20) using Microsoft Excel’s randomisation function. This selection process is detailed in Fig. 1.

### Menu analysis

The menu analysis took a two-pronged approach using the nutrient analysis software Nutritics v6.06 (Nutritics Ltd, Dublin, Ireland) [30] and the Food Standards Agency’s Nutrient Profiling Model (NPM) [31]. The nutrient content analysis in Nutritics provided the macro- and micronutrient composition of each meal, while the Nutrient Profiling Model (NPM) quantifies the overall healthiness of a meal or food item by assigning points to specific nutrients. Prior to nutrient content and NPM analysis, the characteristics of each meal, including the primary component of the main dish, side offerings, and desserts, were tabulated by hand.

### Nutrient content analysis

One menu cycle from each school, spanning 2–4 weeks depending on the caterer, was input into Nutritics to determine the macro- and micronutrient content of each meal. A lunch (or a meal) was based on one main dish plus sides and desserts as provided on the menu. Nutritics uses the GB23 database comprises 2887 foods meticulously compiled from various sources, including the McCance and Widdowson’s Composition of Foods Integrated Dataset (CoFID) 2021 and the 2021 Quadram labelling dataset [32]. The food items from the menu were matched to the closest food items commonly consumed in the UK in the database [33]. Portion sizes were estimated using the upper portion guidelines for primary schools in the UK Department for Education’s Portion Sizes and Food Groups guidance [34]. Details on portion sizes and adjustments for composite dishes can be found in Appendix A. In the event of a missing food item in the Nutritics database or if only a brand-name item was available, an alternative of similar nutritional value was substituted (i.e. chicken sausages substituted for turkey sausages). This analysis only examined main lunch meal offerings, sides, and desserts; designated vegetarian menus were excluded from the analysis. Self-serve salad bars, bread, jacket potato offerings, or drinks served with lunch were excluded because some menus lacked specific details on the type (e.g., white vs. wholemeal bread or milk vs. juice) or quantity (e.g., self-serve options) of items offered.

The reported nutrients included total energy (kcal), macronutrients (carbohydrates, fat, protein, saturated fat, fibre, total sugar, cholesterol), minerals (sodium, iron, zinc, calcium), and vitamins (A, B9, C, D). The examined vitamins and minerals were those essential for childhood development [35] and those identified by the UK Department of Education as common deficiencies among school-age children [20]. Calories from dessert were derived from the nutrient analysis report, and the percentage of total energy coming from carbohydrates, protein, fat, and dessert was calculated in Excel. Comparisons of the individual nutrient contents of lunches between low- and high-poverty boroughs were performed using the mean nutrient contents of the lunch meals provided in 10 schools in each poverty category.

The individual nutrient content of each lunch was also compared to the Scientific Advisory Committee on Nutrition (SACN)’s daily dietary recommendations [36]. Since reference nutrient intakes (RNIs) for males and females ages 4–6, 7–10, and 11–14 are different, a unified RNI (uRNI) for individual nutrients were created via weighting and averaging calculation (Appendix B). As it is generally considered that lunch should comprise 30% to 35% of the total recommended daily intake for each nutrient [37], the uRNIs were multiplied by 0.3 and 0.35 to create a reference value range. The nutrient contents of each lunch were then categorised as below, above or within the range accordingly.

### Nutrient Profiling Model (NPM)

The NPM was developed by the UK Food Standards Agency in 2007 to guide media and communications regulator Ofcom in restricting advertisements of unhealthy food to children [31]. The model uses a scoring system to analyse the nutrient content of a food or drink per 100g. An item is scored on two scales: “A”, which assigns points to nutrients to limit including energy (kJ), saturated fat, sugar, and sodium and “C”, which assigns points to beneficial food or nutrients including fruit and vegetable content, protein, and AOAC fibre. The item’s total “C” points are subtracted from its “A” points to provide the final nutrient profile score; a score greater than or equal to four indicates that an item is “less healthy” and should not be marketed to children [31]. Details on NP score calculation and scoring are detailed in Appendix C.

Main dishes and sides were examined together as one meal and separately from desserts for this portion of the analysis. The nutrient analysis report was first split by the ingredients of each meal component. For each meal and dessert, kilocalories were converted to kilojoules using the conversion rate of 1 kcal = 4.184kJ [38]. Fruit and vegetable content was calculated by dividing the weight of fruit and vegetables in grams by the total meal weight. Fruit- or vegetable-based sauces (i.e.: tomato sauce) did not count toward fruit and vegetable content. The total energy (kcal), saturated fat (g), total sugar (g), protein (g), fibre (g) and sodium (mg) were adjusted to provide values per 100 g of the meal or dessert with reference to the portion size shown earlier. Similarly, the average of individual nutrients in all menus separately in high- and low-poverty boroughs was calculated and compared.

### Childhood obesity across poverty groups

To establish the magnitude of variations in childhood obesity prevalence across high- and low-poverty boroughs of interest, trends in body mass index (BMI) from reception (ages 4–5) to year 6 (ages 10–11) were analysed across poverty categories. Data from NHS England’s National Child Measurement Programme [2] provides the proportion of children within each age- and sex-specific BMI category on a local-authority level. Each school year, the heights and weights of children entering reception and year 6 are recorded by school nurses using approved and calibrated equipment. These measurements are used to calculate individual BMI. They are then reported to the appropriate local authority, providing the prevalence of underweight, healthy weight, overweight, and obesity (including severe obesity) within the borough.

The sample included all children attending a school within the four boroughs of interest who entered reception in 2017 and year 6 in 2023. The proportion of children in each BMI category within the low- and high-poverty boroughs were presented as mean and range. These were then compared within and across poverty groups at reception and year 6 to establish differences in BMI status. Differences in BMI across poverty categories at reception were calculated to determine baseline variations in obesity. From this, the change in each BMI category’s prevalence was calculated and compared to identify trends in children’s BMI status after their time in primary school and exposure to school food.

### Statistical methods

Statistical analysis was performed using the Statistical Package for Social Sciences (SPSS) v29.0 for MacOS. Demographic, economic, and household data were presented as mean and range values for each poverty group in comparison with the characteristics of London overall. Descriptive statistics provided the means and standard deviations of the complete meals for each poverty category. These were also provided to determine the average percentage of children in each BMI category for each poverty group. Categorical data (e.g. meal types) were presented as frequency (*n*) and percentage (%). Continuous data were presented as mean ± standard deviation (SD); given the large sample of meals (*n* = 295) and the lack of skewness displayed in the descriptive statistics (skewness < ± 1), normality was assumed without the use of a Shapiro–Wilk test.

Independent sample t-tests were used to assess individual nutrient differences between poverty groups. Nutrient values in relation to the uRNI range were examined categorically (above, below, or within range) using Chi-squared tests. Odds ratios (ORs) were calculated to determine associations with poverty status and risk of being above or below the reference values. Differences in NPM scores between groups were determined using an independent sample t-test, and a Chi-squared test was used to establish the relationship between poverty status and “less healthy” classification from the NPM.

Analysis of childhood obesity prevalence using the NCMP was limited to only descriptive statistics; given the small sample size of examined boroughs (*n* = 4), hypothesis testing would not be valid as the analysis would be underpowered. The changes in BMI status from reception to year 6 of each poverty group were calculated in Excel using the average percentage of school children within each BMI category.

## Results

### Demographics

Demographic, economic, and household data for the two lowest- and two highest-child-poverty boroughs are displayed in Table 1.

**Table 1:** Average demographic, economic, and household characteristics of the examined boroughs compared to London

	London	Low Child Poverty (*n* = 2)		High Child Poverty (*n* = 2)	
		Mean	Range*	Mean	Range*
Population	9,089,736	241,086	193,137	299,322	65,128
Under 15 (%)	18.7	14.8	1.7	17.5	1.2
Child Poverty (AHC) (%)	34.7	31.0	4.0	46.0	2.0
Ethnicity (%)					
Asian	20.7	11.8	0.2	27.4	34.0
Caribbean/African	13.5	9.0	2.2	14.2	13.8
Multiple Ethnicities	5.7	6.4	0.3	5.8	1.7
White	53.8	65.8	4.1	46.3	13.7
Other	6.3	7.0	5.8	6.3	4.8
Religion (%)**					
None	27.1	30.5	4.1	31.5	9.7
Christian	40.7	45.5	5.8	26.5	8.4
Buddhist	0.9	0.9	0.4	0.9	0.1
Hindu	5.1	1.6	0.9	1.4	1.2
Jewish	1.7	1.2	1.4	3.6	6.3
Muslim	15.0	10.9	1.9	26.6	26.6
Sikh	1.6	0.3	0.1	0.5	0.4
Other	1.0	0.6	0.1	1.2	1.4
Households (%)					
Single Family Household	58.0	50.2	7.9	51.4	0.9
Deprived in ≥ 1 Dimension	51.9	44.5	6.0	54.3	1.0
Owns Home	46.7	38.5	11.6	26.4	1.4
Socially Rented	23.1	23.4	8.3	38.2	4.6
Education (%)					
No qualification	5.2	3.5	1.7	13.1	7.2
Level 3 or Above	74.9	82.2	7.4	75.4	9.3
Employment & Economy					
Median Weekly Pay (£)	767	935.5	39.0	825.5	61.0
Unemployment Rate (%)	5.5	4.7	2.0	5.6	0.6
Receiving Benefits (%)	15.2	11.2	1.9	18.2	1.9

^*^Range was presented as the difference between the two boroughs for each poverty category; **”Not answered” was excluded from the analysis

Boroughs with the highest child poverty rates had larger total populations and higher percentages of the population under 15 years of age. These boroughs also had a higher percentage of Asian and Caribbean/African residents, whereas low-poverty boroughs were over 50% white. High-poverty boroughs were majority Muslim, followed by Christian, while low-poverty boroughs were majority Christian or not religiously affiliated. There was also considerable variation in Muslim, Jewish, and Christian affiliation between the high-poverty boroughs. Household composition was similar across poverty groups, with 1.2% more single-family households in high-poverty boroughs. However, there were large differences in home ownership and socially rented accommodation across groups. Nearly 10% more households in high- poverty boroughs were deprived in at least one dimension.

Further, the percentage of residents with no educational qualifications was nearly four times higher in high-poverty boroughs. Employees living in low-poverty boroughs made £110 more per week on average. Individuals in these boroughs were also less likely to be unemployed or be receiving out-of-work benefits (Table 1).

### Menu analysis

A total of 295 lunches, 150 in low-poverty boroughs and 145 in high-poverty boroughs, were analysed, all including a main course, a side, and a dessert. The characteristics of the meals offered are presented in Table 2.

**Table 2:** Characteristics of examined main, side, and dessert offerings at primary schools within the highest and lowest child poverty Inner London boroughs (Autumn/Winter 2024)

	Low Child Poverty (*n* = 150)		High Child Poverty (*n* = 145)	
	*n*	%	*n*	%
Main Dishes				
Roast Meat or Poultry	15	10%	22	15%
Meat Products (sausages, rolls, pies)	16	11%	9	6%
Composite Meat-Based Dishes (curries, stews, etc.)	42	28%	35	24%
Battered or Breaded Fish	29	19%	25	17%
Plant-Based Meat Alternatives	3	2%	1	1%
Starch-Based Dishes (pizza, pasta, etc.)—with meat	18	12%	16	11%
Starch-Based Dishes (pizza, pasta, etc.)—vegetarian	25	17%	36	25%
Pulse or Vegetable Dishes (dahl, soups, etc.)	2	1%	1	1%
Accompaniments				
Bread and Vegetables	25	17%	21	14%
Rice and Vegetables	16	11%	20	14%
Boiled or Mashed Potatoes and Vegetables	10	6%	14	10%
Roasted or Fried Potatoes and Vegetables	61	41%	59	41%
Vegetables only	38	25%	31	21%
Desserts				
Biscuits or Flapjacks	31	21%	8	5%
Cakes	49	33%	10	7%
Cheese & Crackers	5	3%	21	14%
Custard, Pudding, or Ice Cream	12	8%	4	3%
Fresh Fruit or Fruit Jelly	19	13%	8	5%
Fruit-based Crumble or Pie	6	4%	11	8%
Yogurt (with fruit or granola toppings)	26	17%	82	57%
Other	2	1%	1	1%
Desserts containing Confectionary or Chocolate	14	9%	3	2%

The most common type of main dish in low-poverty boroughs was composite meat-based dishes like curries and stews (28%); in contrast, starch-based vegetarian dishes, such as pizza or pasta, were most common in high-poverty boroughs (25%). Both poverty groups most frequently served roasted or fried potatoes with vegetables as a side dish (41%). Yoghurt with fruit was the most common dessert offering in high-poverty boroughs (57%), while cakes were served most frequently in low-poverty local authorities (33%). Nine per cent of all desserts served in the most affluent areas contained confectionery or chocolate, as opposed to 2% of desserts in high-poverty boroughs (Table 2).

### Nutrient content analysis

Table 3 presents the average energy and nutrient composition of the complete lunch offerings in the most and least deprived boroughs in comparison to the 30–35% uRNI targets for children aged 6–11. Table 3 also includes the caloric share of carbohydrates, fats, protein, fruit or vegetables, and dessert.

**Table 3:** Average nutritional composition of meals between the Inner London boroughs with the highest and lowest child poverty

Nutrients	Reference Values†	Low Child Poverty (*n* = 150)		High Child Poverty (*n* = 145)		p*
		Mean	SD	Mean	SD	
Energy (kcal)	521—608	595.27	114.11	527.91	112.27	< 0.001
Carbohydrates (g)	69—81	69.33	18.13	62.04	18.52	< 0.001
Protein (g)	8—10	26.62	10.91	29.25	8.74	0.012
Fat (g)	20 −24	23.48	7.70	18.02	8.82	< 0.001
Fibre (g)	6.2–7.3	6.90	2.09	6.82	5.93	0.364
Total Sugars (g)	-	20.93	6.81	17.39	5.93	< 0.001
Saturated Fat (g)	6.4–7.4	8.80	4.30	5.95	3.94	< 0.001
Cholesterol (mg)	-	81.98	50.40	59.30	35.28	< 0.001
Sodium (mg)	549—640	529.14	232.40	453.98	253.13	0.004
Calcium (mg)	171—200	198.44	107.31	240.60	105.89	< 0.001
Iron (mg)	2.6–3.0	3.07	1.06	2.60	0.76	< 0.001
Zinc (mg)	2.1–2.5	3.21	2.10	2.75	1.02	0.009
Vitamin A (μg)	146—170	388.92	281.31	301.20	289.28	0.004
Vitamin D (μg)	3.0–3.5	0.51	0.57	0.37	0.87	0.051
Folate (B9) (μg)	43—50	63.83	22.25	62.37	17.26	0.262
Vitamin C (mg)	9—11	26.75	22.52	30.54	15.06	0.045
% Fruit & Vegetables (Main & Side)	-	29.86	9.28	31.01	10.79	0.163
Dessert Calories (kcal)	-	172.53	86.58	136.46	56.42	< 0.001
% Calories from Dessert	-	28.06	11.13	25.37	7.59	0.008
% Calories from Carbohydrates	-	46.61	8.52	47.07	7.10	0.310
% Calories from Protein	-	18.39	8.30	23.22	8.44	< 0.001
% Calories from Fat	-	34.96	8.64	29.56	10.44	< 0.001

**Fig. 2:**
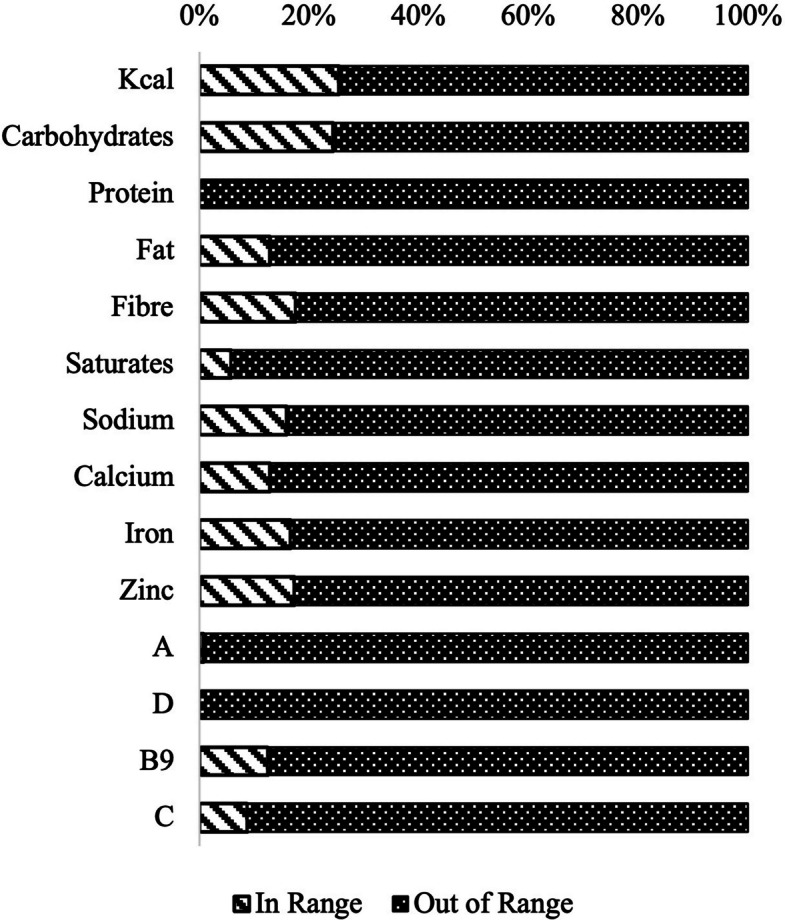
Percentages of all school meals within reference value ranges

**Fig. 3:**
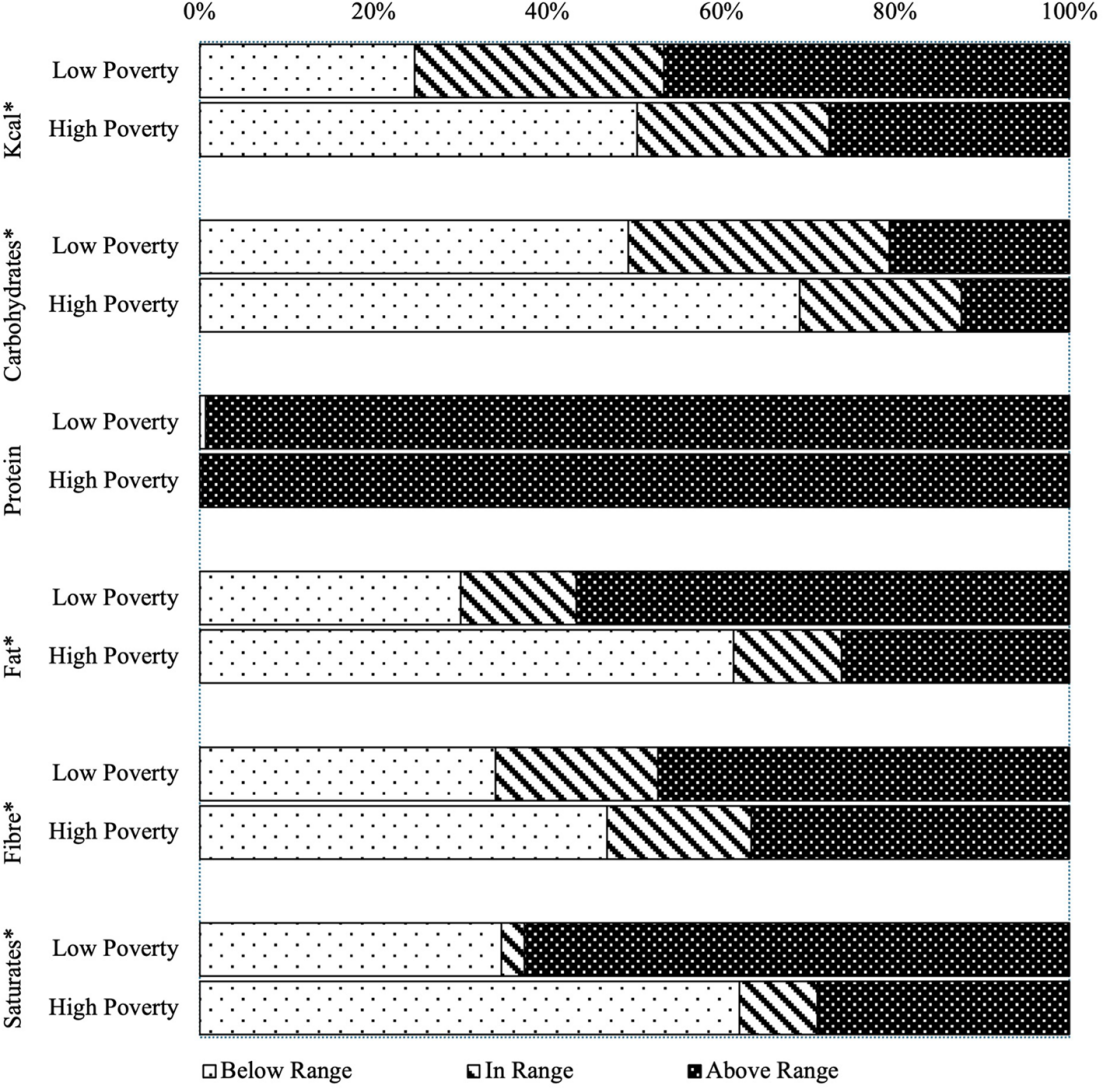
Distribution of macronutrients by borough poverty status with respect to 30–35% uRNI targets. * *p* < 0.05 between the two groups determined with a chi-squared test

See Figs. 2 & 3 for a comparison to reference values and Appendix B for weighted average calculations

^*^Independent t-test

^†^ Reference values calculated using a weighted average of Public Health England’s daily energy and nutrient recommendations for children aged 1–18

Meals in high-poverty boroughs provided significantly fewer total calories, carbohydrates, and total fat on average than low-poverty boroughs (*p* < 0.001). Saturated fat content also varied significantly by poverty status, with high-poverty school meals providing approximately 33% less saturated fat on average (*p* < 0.001). All schools exceeded the reference value range for protein, and high-poverty meals provided an average of 2.6 more grams of protein than low-poverty meals (*p* = 0.012). On average, high-poverty meals contained significantly less cholesterol (*p* < 0.001), sodium (*p* = 0.004), iron (*p* < 0.001), zinc (*p* = 0.009), and vitamin A (*p* = 0.004). In contrast, school meals in the most deprived boroughs were significantly higher in calcium (*p* < 0.001) and vitamin C (*p* = 0.045) (Table 2). The share of total calories coming from dietary fats and protein varied significantly by borough poverty. School meals in high-poverty boroughs had 4.8% more of their total meal calories coming from protein on average and 5.4% less coming from fat than low-poverty school meals (*p* < 0.001). Desserts also had a decreased calorie share in high-poverty school meals, with an average of 36.5 fewer calories (*p* < 0.001) and 2.7% less of the average meal’s total energy (*p* = 0.008) coming from dessert. There was no significant difference in main-course fruit and vegetable content between low and high-poverty boroughs (Table 3).

Only 25.4% of all meals were within the target range for energy, and 24.4% were within the range for carbohydrates. Less than 1/5 of all meals were within range for total fat (12.9%), fibre (17.6%), sodium (15.9%), calcium (12.9%), iron (16.6%), zinc (17.3%), and folate (B9) (12.5%). Less than 10% of all meals were within the uRNIs for saturated fat (5.8%), vitamin A (0.7%), and vitamin C (8.8%). All school meals were out of the reference value range for protein, as well as vitamin D (Fig. 2).

High-poverty school meals were 56% less likely to exceed the reference value range for energy (*p* < 0.001). They were also significantly less likely to exceed the upper reference value for total fat and saturated fat (*p* < 0.001). Compared to high-poverty boroughs, school meals in the most affluent boroughs had four times increased odds of exceeding saturated fat recommendations (*p* < 0.001) (Table 4). All school meals were above the recommended values for protein, regardless of poverty bracket (Fig. 3). High-poverty lunches were two times more likely to be below the reference value range for carbohydrates (*p* < 0.001) (Table 4), with no significant difference in odds of exceeding the recommended range (Table 5). While mean differences in dietary fibre were not significant between low and high-poverty boroughs (Table 3), the distribution of fibre varied significantly by poverty status. 46.9% of high-poverty meals were below the reference value, versus 34% of low-poverty meals (Fig. 3). High-poverty school meals had almost twice the odds of providing less than the recommended serving of fibre (*p* = 0.024) (Table 5).

**Table 4:** Probability of exceeding the reference values by poverty category

Nutrients	Low Child Poverty (*n* = 150)		High Child Poverty (*n* = 145)		p*
	OR	95% CI	OR	95% CI	
Energy (kcal)	2.30	1.41–3.73	0.44	0.27–0.71	< 0.001
Carbohydrates (g)					0.057
Protein (g)					0.325
Fat (g)	3.68	2.2–6.02	0.27	0.17–0.44	< 0.001
Fibre (g)					0.061
Saturated Fat (g)	4.12	2.53–6.71	0.24	0.15–0.40	< 0.001
Sodium (mg)					0.487
Calcium (mg)	0.56	0.35–0.88	1.80	1.14–2.86	0.012
Iron (mg)	2.10	1.30–3.37	0.48	0.30–0.77	0.002
Zinc (mg)	1.65	1.04–2.63	0.61	0.38–0.96	0.033
Vitamin A (μg)	2.11	1.28–3.48	0.47	0.29–0.78	0.003
Vitamin D (μg)					0.057
Folate (B9) (μg)	0.51	0.30–0.86	1.98	1.17–3.34	0.010
Vitamin C (mg)	0.16	0.07–0.38	6.27	2.67–14.52	< 0.001

Non-significant odds ratios were not detailed in the table

^*^Significance of the odds ratio

**Table 5:** Probability of being under the reference values by poverty category

Nutrients	Low Child Poverty (*n* = 150)		High Child Poverty (*n* = 145)		p*
	OR	95% CI	OR	95% CI	
Energy (kcal)	0.32	0.20–0.53	3.10	1.89–5.07	< 0.001
Carbohydrates (g)	0.44	0.27–0.71	2.28	1.42–3.67	< 0.001
Protein (g)					0.325
Fat (g)	0.27	0.17–0.44	3.71	2.29–6.01	< 0.001
Fibre (g)	0.58	0.37–0.93	1.71	1.07–2.74	0.024
Saturated Fat (g)	0.32	0.02–0.52	3.08	1.91–4.96	< 0.001
Sodium (mg)					0.181
Calcium (mg)	2.98	1.81–4.90	0.37	0.20–0.55	< 0.001
Iron (mg)	0.45	0.28–0.73	2.20	1.38–3.53	< 0.001
Zinc (mg)					0.832
Vitamin A (μg)	0.47	0.28–0.78	2.13	1.29–3.52	0.003
Vitamin D (μg)					0.057
Folate (B9) (μg)					0.066
Vitamin C (mg)	3.35	1.06–10.51	0.30	0.10–0.94	0.029

Non-significant odds ratios were not detailed in the table

^*^Significance of the odds ratio

**Fig. 4:**
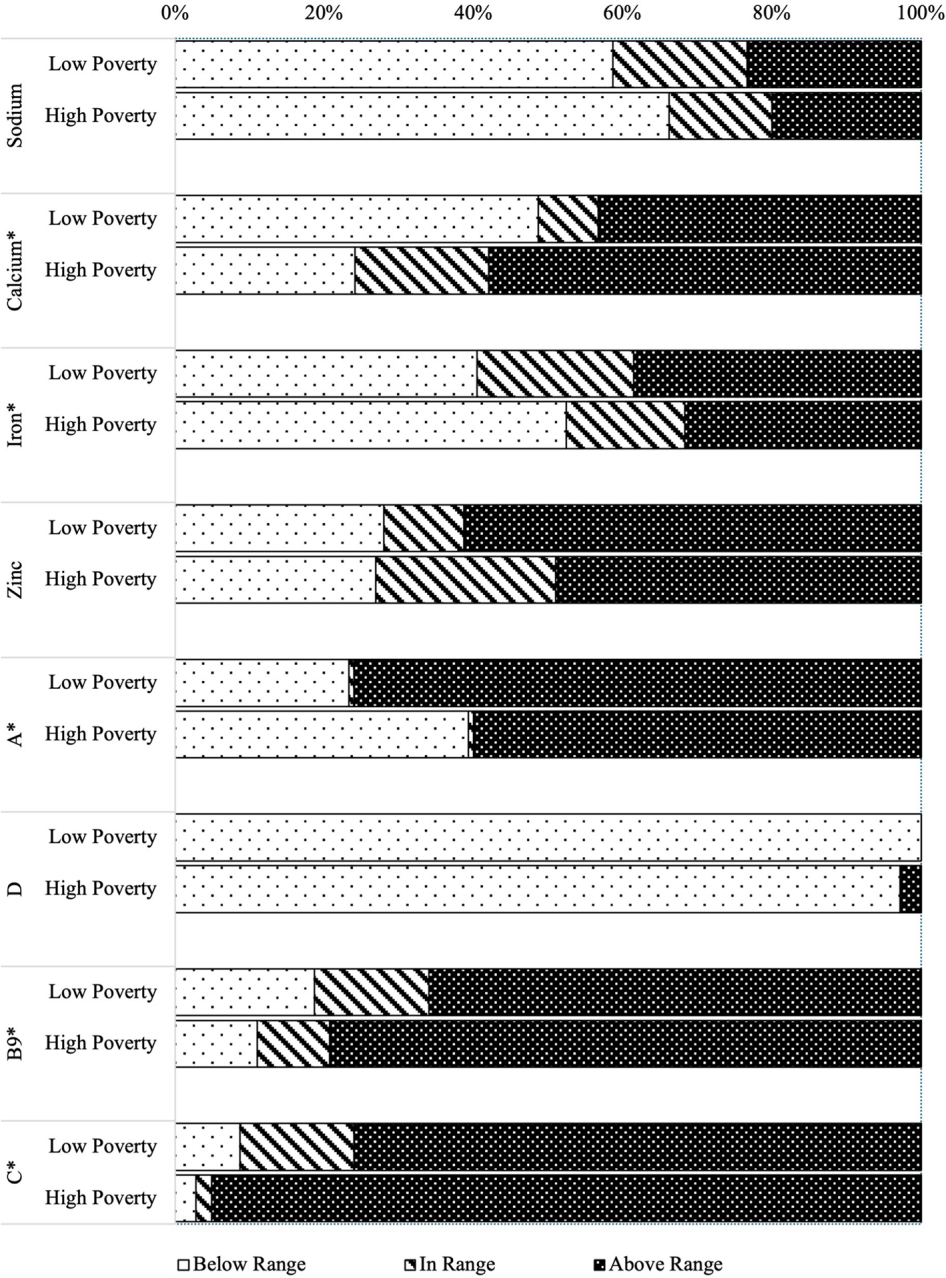
Distribution of micronutrients by borough poverty status with respect to 30–35% uRNI targets*.* * *p* < 0.05 between the two groups determined with Chi-squared test

Sodium consumption in both poverty categories was relatively positive, with approximately 75% of meals below or within reference values (Fig. 4). Poverty status was inversely proportional to calcium, folate (B9), and vitamin C content, with meals in the more deprived schools being two times more likely to exceed the reference value for calcium, two times higher odds of being above range for folate (*p* = 0.010) and six times more likely to exceed the target range for vitamin C (*p* < 0.001). Almost all meals were below the low reference value for vitamin D (Fig. 4).

### Nutrient profiling model

**Fig. 5:**
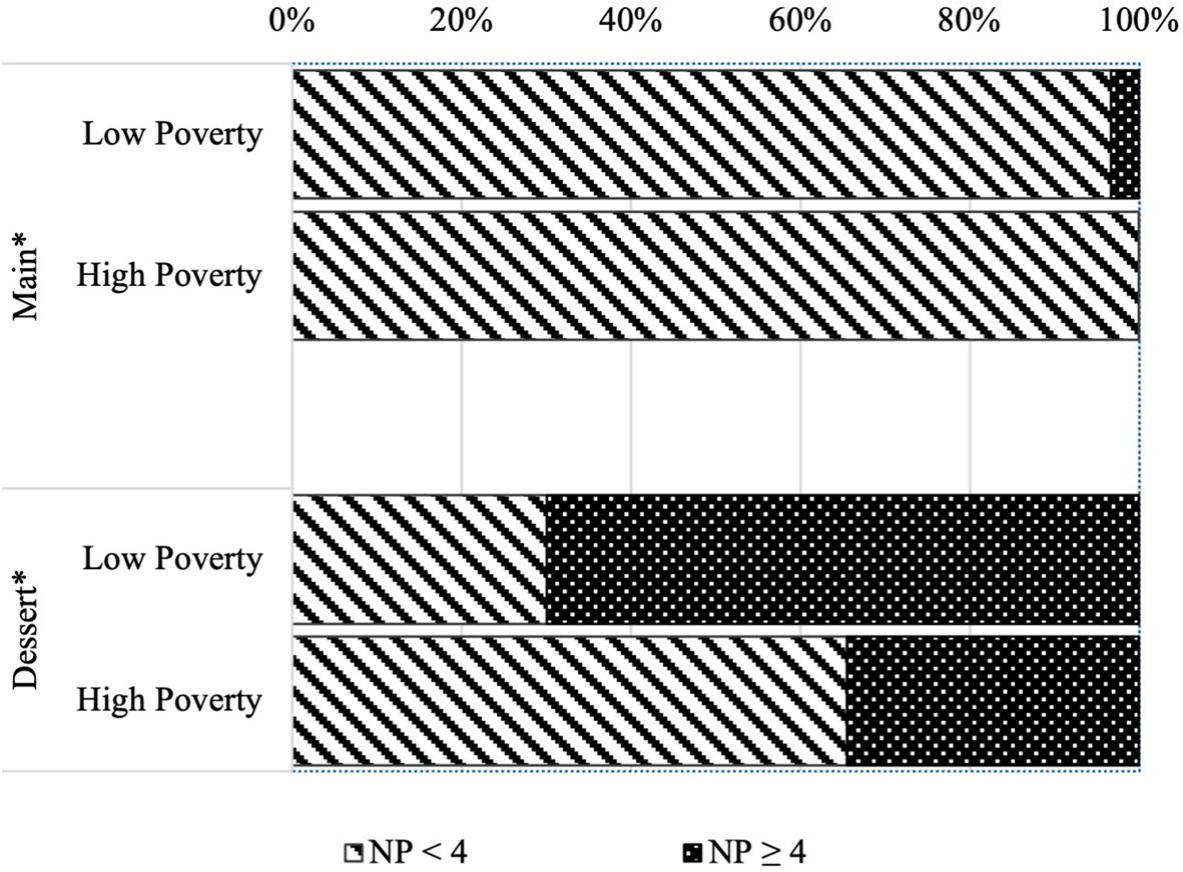
Distribution of NP scores by borough poverty status. *p < 0.05 between the two groups determined by a chi-squared test

Main courses in high-poverty boroughs scored better than low-poverty boroughs, with an average of −2.7 (± 1.98) NP points and −2.2 (± 2.25) NP points, respectively (*p* = 0.016). All high-poverty main dishes scored less than 4 NP points, versus 96.7% of low-poverty mains (*p* = 0.027) (Fig. 5). The same is true for desserts, with high-poverty boroughs scoring 4.5 (± 10.38) NP points and low-poverty boroughs scoring 10 (± 10.25) NP points on average (*p* < 0.001). At the cut-point of NP = 4, both groups’ desserts are considered “less healthy” on average, according to the NPM. However, only 34.5% of desserts in high-poverty boroughs had NP scores of 4 or higher compared to 70% of low-poverty desserts (*p* < 0.001) (Fig. 5).

Desserts with the highest NP scores were rice crispy cakes (NP = 26), sponge cake with chocolate sauce (NP = 25), cheese with breadsticks (NP = 22), and flapjacks (NP = 19). The lowest-scoring dessert offerings were yoghurt with fruit (NP = −4), fruit salad (NP = 2), and fruit crumble (NP = 3). The only main meal that was deemed “less healthy” using the NPM was a sausage roll served with potato wedges, cabbage, and carrots (NP = 4).

### Childhood obesity across poverty groups

Tables 6 present the mean percentage of children entering reception and year 6 in each BMI category within the examined boroughs (*n* = 4). The difference (Δ) between Year 6 and Reception is also detailed within the tables.

**Table 6:** Mean percentage of children within each BMI category entering reception in 2017 and year 6 in 2023

Child poverty categories	Reception (*n* = 2)		Year 6 (*n* = 2)		Δ**
	Mean (%)	Range* (%)	Mean (%)	Range* (%)	(%)
Low Child Poverty (*n* = 2)					
Underweight	1.2	0.3	1.5	0.6	0.3
Normal	78.4	3.1	63.8	0.4	−14.6
Overweight	11.2	0.1	13.4	1.1	2.2
Obese (including severely obese)	9.3	3.4	20.9	0.9	11.6
Severely Obese	2.7	1.4	5.8	0.7	3.1
High Child Poverty (*n* = 2)					
Underweight	2.1	0.6	2.0	0.8	−0.1
Normal	77.1	2.7	57.3	1.6	−19.8
Overweight	10.3	1.8	14.4	0.2	4.1
Obese (including severely obese)	10.5	1.5	26.4	0.6	15.9
Severely Obese	3.3	0.1	8.3	1.5	5

^*^Range was presented as the difference between the two boroughs for each poverty category; **difference = ‘Year 6’-‘Reception’

Despite similar distributions of percentage in different BMI categories across poverty lines at reception, high-poverty boroughs experienced greater increase in obesity from reception to year 6 compared with low-poverty boroughs (15.9% vs 11.6%). In these areas, the percentage of students with normal weight decreased, with a 19.8% decline in high-poverty areas and a 14.6% decline in low-poverty areas. The percentage of children who were overweight rose by 4.1% in the most deprived boroughs, versus a 2.2% increase in the most affluent local authorities. The trend was similar for severe obesity, with an increase of 5% in the most deprived boroughs and 3.1% in the least deprived. Overall, 25.0% of children in the high-poverty boroughs were overweight or obese compared with 16.9% in the low-poverty boroughs.

## Discussion

This analysis of a large sample of school meals across Inner London’s most and least deprived boroughs revealed that, overall, lunch offerings in high-poverty boroughs were objectively healthier than those in the most affluent areas. High-poverty school meals were significantly lower in total energy, fat, carbohydrates, saturated fat, sugar, and cholesterol. However, meals in these areas provided significantly less iron, zinc, and vitamin A, while also having an increased risk of falling below the reference value range for these key micronutrients. Using the nutrient profiling model, high-poverty meals scored significantly better than low-poverty lunches in both main courses and desserts. Despite this, the prevalence of overweight, obesity, and severe obesity is disproportionately higher in children entering year 6 in high-poverty boroughs.

The UK Department for Education identifies iron, zinc, and calcium as key nutrients of concern for school-aged children [20]. High-poverty meals contained significantly less iron and zinc, likely due to a higher prevalence of vegetarian meals, 26% in high-poverty vs. 18% in low-poverty areas (Table 1). Zinc intake is generally lower in plant-based diets [39], as it is mostly sourced from animal products [40]. Although plant-based diets can provide more iron than omnivorous ones [41], the starch-based vegetarian meals common in high-poverty schools often lacked iron-rich plant foods like legumes or spinach [42], and both plant-based iron and zinc are less bioavailable [43]. Conversely, calcium content was significantly higher in high-poverty meals, with a 63% lower risk of deficiency, likely due to frequent servings of yoghurt [44]. In addition, cultural dietary practices and high costs of meat may explain the increased frequency of vegetarian meals in high-poverty boroughs.

On average, desserts served in low-poverty boroughs provided significantly more calories than desserts served in high-poverty boroughs. Further, low-poverty desserts scored more than double the NPM points of desserts in high-poverty areas. This may explain the 20% increase in average total sugar and 33% higher saturated fat content in low-poverty school meals compared to high-poverty lunches. High-poverty schools frequently served desserts like yoghurt with fruit or fruit salad, which is lower in sugar and saturated fat than the cakes and biscuits most offered in low-poverty schools. This may be due to the typically lower costs of catered fruit compared with cakes and biscuits [45], as well as decreased preparation requirements and therefore lower costs [45, 46].

In addition, boroughs with high-child-poverty may be more likely to prioritise school food within wider public health and health inequalities strategies, leading to closer oversight of catering contracts, stronger monitoring of compliance with School Food Standards, and additional support for menu planning and food education. In contrast, in lower-child-poverty boroughs there may be less policy emphasis or investment in strengthening school food environments. However, this hypothesis requires further investigation.

The UK Department for Education’s School Food Standards Practical Guide (SFSPG) is comprised of food-based standards, including weekly limits on items such as deep-fried foods and pastries, as well as weekly targets for wholemeal products and vegetables in an effort to reduce salt, sugar, and fat content, as well as improve micronutrient profiles in school meals [20]. Evidence suggests that combining food-based and nutrient-based standards, those specifying minimum and maximum nutrient values, is more effective at lowering salt, fat, and saturated fat while boosting key micronutrients [47]. Currently, the SFSPG does not include any nutrient-based requirements. Adding such standards, particularly for iron and zinc, could enhance the nutritional quality of school meals and further reduce sugar and fat content.

Current SFSPG regulations on desserts are minimal; desserts such as cakes and biscuits are permitted, with no limitation on serving frequency. The only regulation pertaining to desserts is that they should not contain confectionery, chocolate, or chocolate-coated products. Despite this, 9% of low-poverty school desserts contained chocolate or confectionery. In order to reduce the caloric contribution of desserts, along with the sugar and saturated fat content, more stringent regulations should be placed on sweets served in schools.

Improved school food monitoring is also essential. A 2022 pilot by the Food Standards Agency and Ofsted, the UK school inspection authority, found it feasible to include school meal monitoring in routine inspections, but no further updates have been provided [48]. School meal monitoring has proven effective in reducing child BMI and improving diet quality [9]. Nationwide implementation of such programs is urgently needed due to inconsistent compliance with existing standards.

This study’s findings on childhood obesity and area deprivation are consistent with the existing literature. The 2023–24 National Child Measurement Programme (NCMP) found that obesity rates in year 6 were 2.3 times higher in the most deprived areas [7]. Prior NCMP cycles also found children in these areas nearly twice as likely to be overweight or obese [49]. It is important to note that these studies quantified deprivation using the Index of Multiple Deprivation, while in the current study, child poverty after housing costs was used as an indicator of socio-economic status.

While the relationship between area poverty and childhood obesity established in this study aligns with nationwide trends, there is some variation in results with other smaller-scale NCMP studies. An analysis of NCMP data in Hampshire, UK, over time found no significant relationship between school-based area deprivation and BMI change; instead, residential area deprivation showed stronger links [50]. Hampshire’s lower population density (380.8/km^2^) compared to London’s (5,690/km^2^) [51, 52] may explain the divergence. Lower density could increase the distance between home and school, making residential deprivation more impactful. That study also linked BMI data for individual children from reception to year 6, providing a more precise analysis of changes in BMI status. Further research using this methodology is needed to establish the differences, if any, between residence-based and school-based deprivation’s association with child BMI in Inner London. Despite this, more children of low-SES in Hampshire were classified as overweight or obese at baseline and at follow-up, aligning with the findings of this study.

Despite the better nutritional quality of school meals in high-poverty boroughs, childhood overweight and obesity remain disproportionately high, indicating that factors beyond school food contribute to socioeconomic disparities in adiposity. School lunches account for only one meal on school days, while the majority of children’s food intake is determined by parents or carers. Household food insecurity is strongly linked to childhood obesity, with affected children having five times higher odds of being obese [53]. Additionally, low parental education and limited nutrition knowledge can negatively impact the quality of food consumed at home [54]. Physical inactivity also plays a role; children from low-SES backgrounds engage in less physical activity and more sedentary behaviour, as shown in a study of 8–11-year-olds [55]. These findings suggest that broader socioeconomic and behavioural factors may have a greater influence on childhood obesity than school meals alone and partly explain the higher obesity prevalence observed in the highest child poverty boroughs compared with the lowest child poverty boroughs (refer to Table 1). In addition, higher proportion of Black ethnic populations in the high-poverty boroughs (14.2% vs 9.0% in low-poverty boroughs) contributes to the higher childhood obesity prevalence because Black African children were the most likely out of all ethnic groups to be overweight or living with obesity, among both 4 to 5 year olds (27.7%) and 10 to 11 year olds (47.7%) compared with White Caucasian children (22.4% and 34.9% respectively) [56].

This study has notable strengths, particularly in its analysis of school meal nutrition across different poverty classifications. Past studies of school meals in the UK and abroad have examined meal nutritional content holistically; to our knowledge, this is the first study to stratify the results by area deprivation. Furthermore, the use of multiple approaches to evaluate nutritional differences between groups provided insight into the macro- and micronutrient composition of meals and desserts, as well as their healthiness according to the NPM.

There are limitations of the study. Firstly, recipes, ingredient specifications, and actual portion size or weights were unavailable, therefore, the results represent estimated nutrient provision rather than actual nutrient content or intake. More precise analysis would require recipes and portion sizes from involved schools for accurate nutrient content in school meals, or primary data collection, including weighed or estimated food diary recording, to account for food waste and over- or under-serving for accurate nutrient intake. Secondly, bias may be introduced through school inclusion criteria of size which may not be representative of all primary schools in the chosen boroughs. Thirdly, since analysis was based on available online menus, it is likely that schools providing more nutritious lunches are more likely to publish menus online, which may limit the external validity. Furthermore, firsthand observation would have allowed for the inclusion of supplementary meal offerings, such as self-serve jacket potatoes and salad bars specified in some schools’ menus. By solely examining the menus posted online, it is unknown whether these supplementary foods are consumed alongside or in lieu of main menu offerings. Additionally, beverage offerings were also frequently missing from online menus and excluded from analysis to reduce the risk of underestimating nutrients like calcium (from milk) or sugar (from juice). Free sugar content was not assessed due to incomplete data in the Nutritics database, limiting the ability to distinguish between added and naturally occurring sugars or compare values to SACN’s daily limits. Lastly, the use of borough-level NCMP data did not allow for hypothesis testing; individual-level BMI data would be necessary to determine statistically significant differences in obesity prevalence between poverty groups.

Nevertheless, this study provides valuable insight into school meal nutrition across socioeconomic strata in Inner London. While high-poverty meals performed better nutritionally, the higher prevalence of childhood obesity in these areas suggests that broader social and behavioral factors play a more significant role in obesity risk than school meal quality alone.

## Supplementary Information

Supplementary Material 1. Appendix A: Portion Recommendations from the UK Department of Education.

Supplementary Material 2. Appendix B. Weighted mean calculation of 30-35% reference value ranges for the average primary school child.

Supplementary Material 3. Appendix C. Nutrient Profiling Technical Guidance.

## Data Availability

The datasets used and/or analysed during the current study are available from the corresponding author on reasonable request.
