# Supplementary material for: A comparative analysis of primary school meal nutrition across the low- and high-poverty boroughs of Inner London
Source: BMC Nutr. 2026 Mar 18;12:80. doi: 10.1186/s40795-026-01280-w (PMC13112842; doi:10.1186/s40795-026-01280-w)
Supplement: Supplementary file 1 — Supplementary Material 1. Appendix A: Portion Recommendations from the UK Department of Education. [file 40795_2026_1280_MOESM1_ESM.pdf]

Appendix A: Portion Recommendations from the UK Department of Education

Food Category	Modifiers	Upper Portion	Notes
		Starches	
Bread		70g	
Potatoes	Boiled/Mashed	170g	
	Jacket/Baked	280g	
	Roasted/Fried	100g	
Other Starchy Root Vegetables		150g	
Pasta & Noodles		65g dried	
Rice		55g dried	
Other Grains		60g	
Pizza Base		70g	Combined with toppings for total weight. When served alongside another starch, portion was halved.
		Fruits and Vegetables	
Vegetables (excluding below)		60g	Vegetable portion halved in combination dishes where additional vegetables are served as a side.
Pulses		20g dried	
Baked Beans		70g	
Vegetable-based Soup		250g	
Fruits	Large	100g (with skin)	
	Medium	100g (with skin)	
	Small	60g	
	Dried	30g	
		Meats and Sources of Protein	
Red Meat	Roasted	80g	
	Sausages	75g	
	Burgers (patty)	80g raw	"Average Portion" in Nutritics v6.06 used for complete burger with bun and toppings.
	Pies/Rolls	80g total	
Poultry	Roasted	85g	
	Breaded	70g	
Meat-based Soups		250g	
Fish	White	90g	
	Oily	80g	
	Breaded/Battered	80g	

	In Salad or Potato	70g	
Meat Alternatives		70g	
Milk & Dairy			
Milk	Drinking (semi-skimmed)	200ml	
	Puddings	120g	
	Custard	100g	
Yoghurt		120g	
Cheese		30g	
Desserts			
Fruit Jelly		100g	
Cakes and Tray Bakes		50g	
Biscuits and Flapjacks		30g	
Ice Cream		80g	
Sauces and Accompaniments			
Condiments		10g	
Gravy		30g	Also portion used for sauces in composite dishes (i.e: curries, pasta dishes)
Garlic Bread		20g	
Breadsticks and Crackers		15g	
