# Supplementary material for: A comparative analysis of primary school meal nutrition across the low- and high-poverty boroughs of Inner London
Source: BMC Nutr. 2026 Mar 18;12:80. doi: 10.1186/s40795-026-01280-w (PMC13112842; doi:10.1186/s40795-026-01280-w)
Supplement: Supplementary file 2 — Supplementary Material 2. Appendix B. Weighted mean calculation of 30-35% reference value ranges for the average primary school child. [file 40795_2026_1280_MOESM2_ESM.pdf]

Appendix B. Weighted mean calculation of 30-35% reference value ranges for the average primary school child

	Age 4-6 (Reception, Y1)			Age 7-10 (Y2, Y3, Y4)			Age 11-14 (Y5)			**Weighted Mean (x̄)†**	**30% RDI**	**35% RDI**
Weight (*w*)	*w* = 2			*w* = 4			*w* = 1					
Gender (M/F)	M	F	AVG	M	F	AVG	M	F	AVG			
Energy (kcal)	1482	1378	1430	1817	1703	1760	2500	2000	2250	**1736**	**521**	**608**
Carb. (g)	198	184	191	242	227	234.5	333	267	300	**231**	**69**	**81**
Protein (g)	19.7	19.7	19.7	28.3	28.3	28.3	42.1	41.2	41	**27.8**	**8**	**10**
Fat (g) [Less than]	58	54	56	71	66	68.5	97	78	87.5	**68**	**20**	**24**
Fibre (g)	20	20	20	20	20	20	25	25	25	**21**	**6.2**	**7.3**
Sugars (g)*	-	-	-	-	-	-	-	-	-	**-**	**-**	**-**
Saturated Fat (g) [Less than]	18	17	17.5	22	21	21.5	31	24	27.5	**21**	**6.4**	**7.4**
Cholesterol (mg)*	-	-	-	-	-	-	-	-	-	**-**	**-**	**-**
Sodium (mg)	1200	1200	1200	2000	2000	2000	2400	2400	2400	**1829**	**549**	**640**
Calcium (mg)	450	450	450	550	550	550	1000	800	900	**571**	**171**	**200**
Iron (mg)	6.1	6.1	6.1	8.7	8.7	8.7	11.3	14.8	13.05	**8.6**	**2.6**	**3.0**
Zinc (mg)	6.5	6.5	6.5	7	7	7	9	9	9	**7.1**	**2.1**	**2.5**
Vitamin A (µg)	400	400	400	500	500	500	600	600	600	**486**	**146**	**170**
Vitamin D (µg)	10	10	10	10	10	10	10	10	10	**10**	**3.0**	**3.5**
Folates (B9) (µg)	100	100	100	150	150	150	200	200	200	**143**	**43**	**50**
Vitamin C (mg)	30	30	30	30	30	30	35	35	35	**31**	**9**	**11**

**reference values not provided by SACN*

† *calculated using the formula:* $\overline{x} = \frac{\sum wx}{\sum w}$

w = the weight of each data point
x = the value of each data point
