# Supplementary material for: A comparative analysis of primary school meal nutrition across the low- and high-poverty boroughs of Inner London
Source: BMC Nutr. 2026 Mar 18;12:80. doi: 10.1186/s40795-026-01280-w (PMC13112842; doi:10.1186/s40795-026-01280-w)
Supplement: Supplementary file 3 — Supplementary Material 3. Appendix C. Nutrient Profiling Technical Guidance. [file 40795_2026_1280_MOESM3_ESM.pdf]

Appendix C. Nutrient Profiling Technical Guidance

"A" Points

Points	Energy (kJ)	Saturated Fat (g)	Sugar (g)	Sodium (mg)
0	≤ 335	≤ 1	≤ 4.5	≤ 90
1	>335	>1	>4.5	>90
2	>670	>2	>9	>180
3	>1005	>3	>13.5	>270
4	>1340	>4	>18	>360
5	>1675	>5	>22.5	>450
6	>2010	>6	>27	>540
7	>2345	>7	>31	>630
8	>2680	>8	>36	>720
9	>3015	>9	>40	>810
10	>3350	>10	>45	>900

"C" Points

Points	Fruit and Vegetable Content (%)	AOAC Fibre (g)	Protein (g)*
0	≤ 40	≤ 0.9	≤ 1.6
1	>40	>0.9	>1.6
2	>60	>1.9	>3.2
3	-	>2.8	>4.8
4	-	>3.7	>6.4
5	>80	>4.7	>8.0

**a food cannot score points for protein if it scores 11 or more "A" points*
